# Carrageenan from *Gigartina skottsbergii*: A Novel Molecular Probe to Detect SARS-CoV-2

**DOI:** 10.3390/bios13030378

**Published:** 2023-03-13

**Authors:** Patrícia Daiane Zank, Milena Mattes Cerveira, Victor Barboza dos Santos, Vitor Pereira Klein, Thobias Toniolo de Souza, Danielle Tapia Bueno, Tais Poletti, Amanda Fonseca Leitzke, Janice Luehring Giongo, Neftali Lenin Villarreal Carreño, Andrés Mansilla, Maria Soledad Astorga-España, Claudio Martin Pereira de Pereira, Rodrigo de Almeida Vaucher

**Affiliations:** 1 Department of Chemical, Pharmaceutical, and Food Sciences, Microorganism Biochemistry and Molecular Biology Research Laboratory, (LAPEBBIOM), Federal University of Pelotas, Pelotas 96010-610, RS, Brazil; 2 Department of Chemical, Pharmaceutical, and Food Sciences, Laboratory for Lipidomic and Bio-Organic Research, Bioforensic Research Group, Federal University of Pelotas, Pelotas 96010-610, RS, Brazil; 3 Materials Science and Engineering Graduate Program, Technology Development Center, Novonano Laboratory, Federal University of Pelotas, Pelotas 96010-610, RS, Brazil; 4 Antarctic and Subantarctic Macroalgae Laboratory, Universidad de Magallanes, Punta Arenas 01855, Chile; 5 Department of Science and Natural Resources, Magallanes Region and Chilean Antarctic, University of Magallanes, Punta Arenas 01855, Chile

**Keywords:** COVID-19, macroalga, carrageenan, probe, qRT-PCR, RT-LAMP

## Abstract

The COVID-19 pandemic has caused an unprecedented health and economic crisis, highlighting the importance of developing new molecular tools to monitor and detect SARS-CoV-2. Hence, this study proposed to employ the carrageenan extracted from *Gigartina skottsbergii* algae as a probe for SARS-CoV-2 virus binding capacity and potential use in molecular methods. *G. skottsbergii* specimens were collected in the Chilean subantarctic ecoregion, and the carrageenan was extracted —using a modified version of Webber’s method—, characterized, and quantified. After 24 h of incubation with an inactivated viral suspension, the carrageenan’s capacity to bind SARS-CoV-2 was tested. The probe-bound viral RNA was quantified using the reverse transcription and reverse transcription loop-mediated isothermal amplification (RT-LAMP) methods. Our findings showed that carrageenan extraction from seaweed has a similar spectrum to commercial carrageenan, achieving an excellent proportion of binding to SARS-CoV-2, with a yield of 8.3%. Viral RNA was also detected in the RT-LAMP assay. This study shows, for the first time, the binding capacity of carrageenan extracted from *G. skottsbergii*, which proved to be a low-cost and highly efficient method of binding to SARS-CoV-2 viral particles.

## 1. Introduction

SARS-CoV-2 infection (i.e., COVID-19) was declared a pandemic by the World Health Organization (WHO) on March 11, 2020 [1], causing significant epidemiological, social, economic, cultural, and political repercussions [2]. Today, COVID-19 still has a devastating impact, according to the latest WHO report, with over 640 million cases and 6.6 million deaths worldwide [3].

COVID-19 symptoms generally occur 2 to 14 days after infection. Screening these patients is critical for managing viral contamination as it is a rapidly transmitting disease with no cure. Hence, detecting isolated samples from infected patients is the most effective way to contain its spread [4], especially since early diagnosis is crucial in evaluating suspected cases and for determining contagion levels [5]. Real-time reverse transcription polymerase chain reaction (RT-qPCR) has been the global standard for viral identification since the onset of the COVID-19 pandemic. This technique has been highly beneficial in diagnosing and surveilling suspected patients. However, alternative methods to identify SARS-CoV-2 have been proposed [6]. In the last two years, research has sought to develop new molecular strategies for detecting the virus. Recently, a new strip test-based quantitative molecular lateral flow assay for SARS-CoV-2 was developed based on a nucleic acid lateral flow assay of gold nanoparticles [7]; it proved to be a straightforward and inexpensive method with quick analysis and simultaneous quantification, providing high levels of detection and specificity.

Effective diagnostics that can rapidly and inexpensively diagnose COVID-19 are urgently needed. Given this concerning scenario, researchers worldwide have sought to develop a fast and easy-to-operate method to detect SARS-CoV-2 [8]. Natural constituents with probes for the binding capacity of the SARS-CoV-2 virus and the use of molecular methods are vital to become a powerful assessment tool [9]. Carrageenan, an algae-derived polymer used in pharmaceutical formulations, is one of these potential natural components. Its bioactive and physicochemical properties make it a promising biomaterial [10] for suspensions, thickeners, and stabilizers. The presence of sulfate esters in the molecule provides desirable properties for numerous applications, including gelling agents and water-holding capacity [11]. For instance, Lobregas, Bantang, and Camacho (2019) [12] developed an Hg^2+^ selective colorimetric detection method employing carrageenan as both a reducing agent and a stabilizer as part of a probe kit with silver nanoparticles.

Carrageenan is also rich in specific sulfated polysaccharides, with a significant effect attributed to their strong ability to interfere with the initial attachment of the virus to target cells and host tissues [13,14]. Kwon et al. (2020) [15] demonstrated that carrageenan binds tightly to the S protein of SARS-CoV-2 in vitro, suggesting that they may act as bait to interfere with the binding of protein S to the sulfate co-receptor in host tissues, inhibiting viral infection. This interaction may occur in the presence of negatively charged sulfate groups, which can neutralize the virus’s positively charged glycoproteins, resulting in a stable virion–carrageenan complex that prevents the virus from completing the infection process [16].

The seaweed *Gigartina skottsbergii*, class *Rhodophyceae*, also known as red algae, can be found in Argentina, Chile, and along the Antarctic Peninsula [17] and it is a rich natural source of carrageenan [18,19]. It is one of the most utilized seaweeds in Chile due to its high carrageenan content, representing over 70% of its dry weight [20]. Our research team previously investigated the biotechnological potential of subantarctic algae, such as lipidomics, and, more recently, using κ-carrageenan as a biodegradable film to activate natural oils with antimicrobial protection [21,22]. In addition, new and pioneering approaches to remove SARS-CoV-2 from water and sewage have been developed, along with alternative methods of diagnosing this virus in healthcare workers [2,6,23,24]. In this study, we aimed to extract and characterize carrageenan from the Chilean macroalgae *G. skottsbergii* for use in molecular methods as a binding probe for the SARS-CoV-2 virus. Our findings revealed, for the first time, that macroalgae have the potential for future applications in detecting SARS-CoV-2.

## 2. Materials and Methods

### 2.1. Materials

#### 2.1.1. Sampling

*G. skottsbergii* specimens were collected in January 2017 in the high meso-littoral zone of the San Juan region (53°43′ S, 70°58′ W) in the Chilean Subantarctic ecoregion. The specimens were packed in thermal boxes filled with seawater and sanitized to eliminate epiphytes. The samples were dried in a JOST 700T air oven at 35 °C for 24–30 h and sprayed in a refrigerator model 226/2 (Lucadema, Brazil). Identification and classification were made, and the samples were placed in a freezer at -20 °C in dark plastic bags [22,25].

#### 2.1.2. Materials

Commercially sourced carrageenan was obtained from Sigma Aldrich^®^, catalogued as C1013 (Kappa carrageenan). Primers were obtained from IDT Biotechnology (Coralville, IO, USA). Prof. Dr. Edison Luiz Durigon from the Microbiology Department, Institute of Biomedical Sciences, University of São Paulo (USP), Brazil, kindly provided the inactivated SARS-CoV-2 (SP02/human 2020/Br, GenBank accession number MT126808.1) virus.

### 2.2. Methods

#### 2.2.1. Carrageenan Extraction

**Figure 1:**
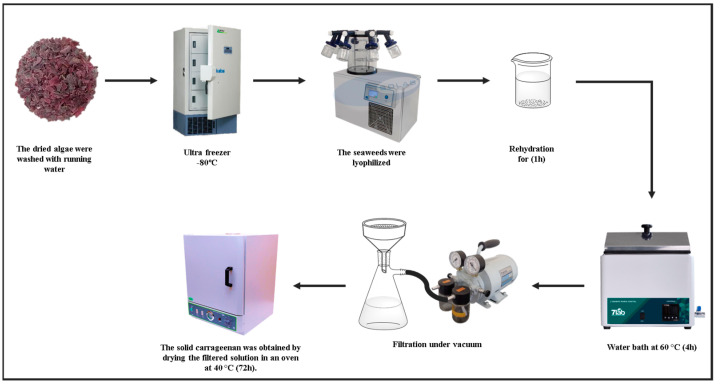
The carrageenan from *G. skottsbergii* graphical extraction process.

Carrageenan was extracted from a sample of *G. skottsbergii*. The extraction was performed according to Webber et al. (2012) [26], with modifications. The previously dried algae were briefly rinsed in running water to remove salts and dirt before being freeze-dried. Ten grams of the sample were weighed and soaked in distilled water (800 mL) for 1 h for rehydration. The solution was then placed in a water bath at 60 °C for 4 h. Filtration was conducted in a vacuum system using filter paper as the filter material to separate the carrageenan from the residue. The solid carrageenan was obtained by drying the filtered solution in an oven at 40 °C for 72 h. A schematic of the extraction process is illustrated in Figure 1.

#### 2.2.2. UV-Vis Scanning Spectroscopy

UV-vis scanning spectroscopy was performed in a Pro-Analysis UV-1600 spectrophotometer with data scanning between 290 and 500 nm using a 1 cm quartz cuvette. Then, 0.5 g of previously extracted carrageenan was solubilized in 100 mL of absolute ethanol under magnetic stirring at 37 °C for 40 min. Aliquots of 1.0 mL were collected and transferred into a volumetric flask containing 24 mL of ethanol; the final concentration was 0.2 mg/mL. The tests were performed in duplicate, and commercial κ-carrageenan (Sigma) was used as a control.

#### 2.2.3. (2,2′-Azino-bis(3-Ethylbenzothiazoline-6-Sulfonic Acid)) ABTS Assay

The ABTS assay was performed according to a previous protocol from our research group [27], adapted from Re et al. (1999) [28]. A 7 mM ABTS^•+^ solution was prepared with a 2.45 mM sodium persulfate solution in PBS (pH 7.4). The solution was allowed to react in the dark at room temperature for 12–16 h before use. The ABTS^•+^ solution was diluted in phosphate-buffered saline (PBS) (pH 7.4) until the absorbance reached 0.700 ± 0.02 at 734 nm. The test compounds were serially diluted two-fold in PBS (500–7.uM). For every 3 mL of the serially diluted test compound, 1 mL of ABTS^•+^ solution was added and allowed to react for 30 s before measuring the absorbance at 734 nm. PBS was used as the negative control and Trolox as the positive control (4.5 mg/mL). The test compounds’ ABTS^•+^ scavenging activity was calculated using the following equation:

% Scavenging activity = [100 − (Abs_sample_ − Abs_blank_)/Abs_abts_] × 100)]

where Abs_sample_ was the absorbance from the compound solution, ABS_blank_ was the absorbance for the negative control, and Abs_abts_ was the absorbance for the PBS containing ABTS^•+^ only.

#### 2.2.4. 2,2-Diphenyl-1-Picrylhydrazyl (DPPH) Assay

The antioxidant activity was assessed by DPPH assay, according to Choi et al. (2002) [29]. The compounds were serially diluted in 2.5 mL of ethanol, and 1 mL of a 0.3 mM DPPH solution was added to each concentration. The samples were allowed to react at room temperature in the dark for at least 40 min, followed by reading the absorbance of the mixture at 518 nm. Absolute ethanol was used as the negative control and Trolox (5.68 mg/mL) as the positive control. The DPPH scavenging activity was calculated using the following equation:

% Scavenging activity = [100 − (Abs_sample_ − Abs_blank_)/Abs_dpph_] × 100)]

where Abs_sample_ was the absorbance from the compound solution, ABS_blank_ was the absorbance for the negative control, and Abs_dpph_ was the absorbance for the ethanol containing DPPH only.

#### 2.2.5. Attenuated Total Reflectance Fourier Transform (ATR-FTIR) Spectroscopy

The ATR-FTIR spectroscopy was performed using the IR model Spirit (Shimadzu, Kyoto, Japan). Commercial κ-carrageenan (Sigma) and carrageenan extracted from the macroalgae were scanned at 400–4000 cm^−1^, 100 scans, transmittance mode, and 4 cm^−1^ resolution.

#### 2.2.6. Qualitative High-Performance Liquid Chromatography (HPLC)

Analyses of commercial carrageenan (Sigma Aldrich) and extracted carrageenan were performed by chromatographic analysis using a method adapted from Navikaite [30] (Thermo Scientific UltiMate 3000 UHPLC system, Waltham, MA, USA). The mobile phase of isocratic elution comprised a mixture of water and formic acid (0.01%) (91.5:8.5). At the same time, solution B was a mixture of water, acetonitrile, methanol, and formic acid (0.01%) (41.5:22.5:22.5:8.5). The analysis was conducted at 30 °C for 5 min, with an injection volume of 10 mg, a flow rate of 1.0 mL/min, and a detection wavelength of 535 nm. A C18 column (Ascentis Express 5 μm—Fused-Core^®^) was coupled to a UV-Vis detector.

#### 2.2.7. Carrageenan As a Binding Probe for SARS-CoV-2

Carrageenan (10 mg) was dried at 37 °C for 24 h and transferred to a microtube containing 1.5 mL of ultra-purified water (free of RNAse enzymes). Then, 150 µL of the inactivated SARS-CoV-2 viral suspension (2.5 × 10^6^ copies/mL) was added, followed by incubation with shaking at 200 rpm and 28 °C for 24 h. Subsequently, the supernatant and adsorbent were removed and placed in another microtube, and the viral RNA was extracted.

#### 2.2.8. RNA Extraction

The SARS-CoV-2 viral RNA was extracted from carrageenan after a binding assay using a MagMax™ Core Nucleic Acid Purification kit (Thermo Fisher Scientific, Waltham, MA, USA). The extracted RNA was quantified by NanoDrop^®^ (Thermo Scientific, Waltham, MA, USA). Approximately 10 ng of RNA was used to perform the RT-qPCR detection.

#### 2.2.9. Real-Time Reverse Transcription PCR (qRT-PCR)

The primer and probe used in the PCR reactions were designed according to the Center for Disease Control and Prevention [31]. A reaction of 25 μL (final volume) was used, with the subsequent volumes added to the 1× concentrated master mix: 5 μL of sample RNA, 12.5 μL of 2× reaction buffer, 1 μL of Superscript^TM^ III One-Step with Platinum^TM^ Taq DNA Polymerase (Invitrogen, Darmstadt, Germany), 0.4 mM of each dNTP, 0.4 μL of a 50 mM MgSO4 solution (Invitrogen), 1 μg of non-acetylated bovine albumin (Roche), 10 μM of each primer 2019-nCoVN1-F2019-nCoV N1 (5′GACCCCAAAATCAGCGAAAT3′), 2019-nCoVN1-R2019-nCoV N1 (5′TCTGGTTACTGCCAGTTGAATCTG3′), 2019-nCoVN1-P2019-nCoV N1 probe (5′-FAM—ACCCCGCATTACGTTTGGTGGACC– BBQ 3′), and DEPC water. The reaction began at 55 °C for 10 min for reverse transcription, followed by 95 °C for 3 min, 40 cycles of 95 °C for 15 s, and 58 °C for 30 s (7500 Real-Time PCR System, Thermo Fisher Scientific, Waltham, MA, USA).

#### 2.2.10. Reverse Transcription Loop-Mediated Isothermal Amplification (RT-LAMP)

The RT-LAMP assay was carried out on isolated RNA after binding the carrageenan probe and SARS-CoV-2 virus particles. The RT-LAMP assay was performed according to Trassante [6]. A reaction mix with a final volume of 25 μL was prepared using the WarmStart^®^ Colorimetric LAMP 2X Master Mix Kit according to the manufacturer’s instructions. Park et al. [32] proposed employing the oligonucleotides for the N gene (Nsp3_1-61). The concentrations in each Nsp3_1-61 oligonucleotide reaction were 0.8 μM of F3 and B3, 0.4 μM of FIP and BIF, and 0.2 μM of LF and LB. Following the preparation of the reaction mixtures, the tubes were placed in a thermal block at 65 °C for 30 min before measurement.

### 2.3. Statistical Methodology

Data for duplicates are expressed as the mean and standard deviation for each experimental point and performed in duplicates. One-way variance analysis (ANOVA) was used to analyze the data, followed by Tukey tests with a significance level of 5%.

## 3. Results and Discussion

### 3.1. Carrageenan Extraction Process

**Figure 2:**
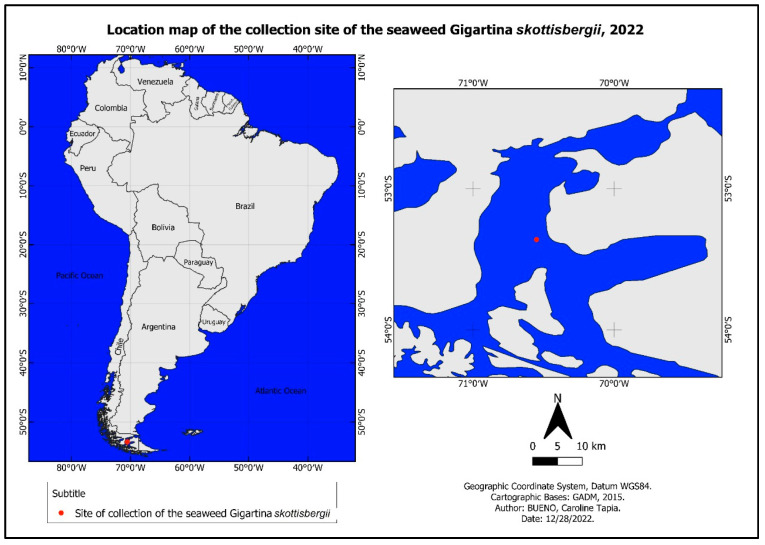
Location of the *G. skottsbergii* seaweed collected.

The *G. skottsbergii* biomass was collected, as with other algae recently studied [33], in the Chilean subantarctic region in the Strait of Magellan in Puerto del Hambre (53°36′ S, 70°55′ W; Figure 2).

**Figure 3:**
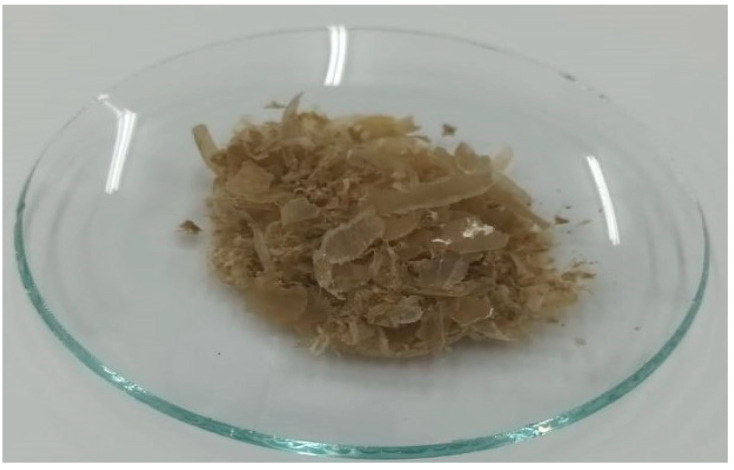
Sample of carrageenan extracted from the red macroalgae *G. skottsbergii*.

Optimum parameters were obtained for the time and extraction temperature of the *G*. *skottsbergii* macroalgae using Webber’s adapted method [26]. The obtained material consistency in Figure 3 suggest that the models are well-adjusted.

### 3.2. UV-Vis Scanning Spectroscopy

**Figure 4:**
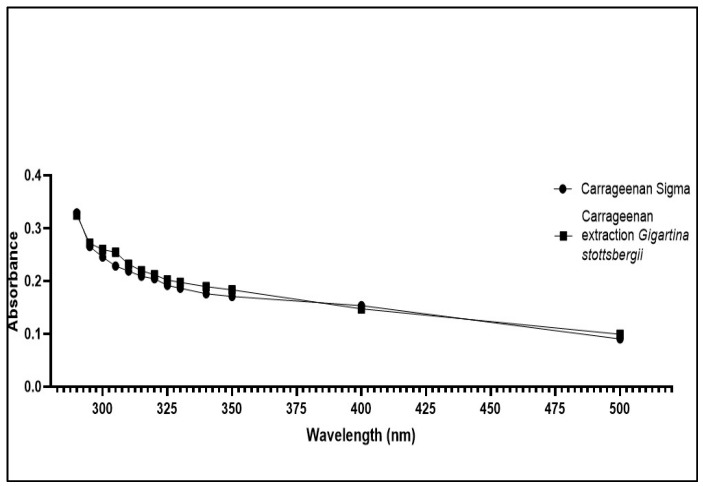
Absorption spectra of carrageenan extraction from the macroalgae *G. skottsbergii* and Sigma^®^ κ-carrageenan (standard).

The UV spectrophotometric analysis detected carrageenan extracted from the macroalgae *G. skottsbergii.* A comparison of the extract’s UV spectrum to that of κ-carrageenan (Sigma^®^) is provided in Figure 4. Their similarity is evident, as no visible peak is shown in the 300–500 nm range, corroborating data reported elsewhere [34,35,36].

### 3.3. Radical Scavenging Capacity

The antioxidant activity of test compounds was evaluated using the 2,2-azino-bis (3-ethylbenzothiazoline-6-sulfonic acid) (ABTS) assay and 2,2-diphenyl-1-picrylhydrazyl (DPPH) radical scavenging capacity.

**Figure 5:**
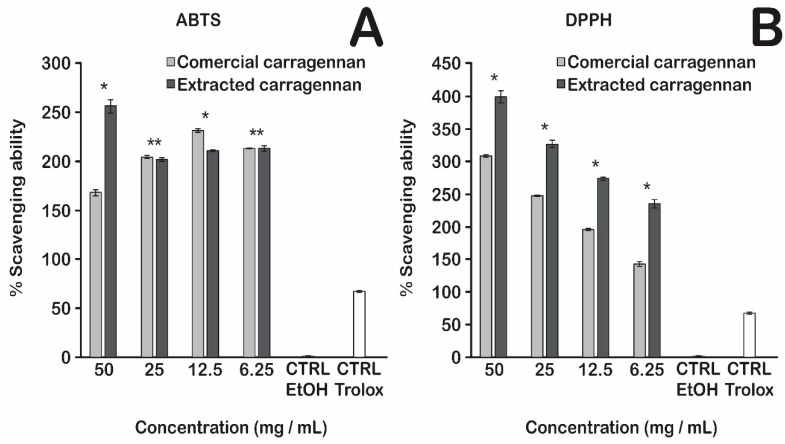
(**A**) ABTS and (**B**) DPPH assays of *G. skottsbergii* seaweed carrageenan extraction and sigma κ-carrageenan; (*) indicate a difference and (**) indicate no statistical difference.

Our results revealed that the carrageenan extraction from the macroalgae *G. skottsbergii* was successfully compared to commercial carrageenan (Figure 5A,B), in which very similar values may be achieved at different concentrations. Both outcomes were compared with the positive control group (Trolox), demonstrating that the experimental carrageenan has a much greater antioxidant capacity than the control group. Moreover, our findings indicate that their values are similar despite the carrageenan extracted from the macroalgae *G. skottsbergii* having a lower antioxidant potential than the commercial carrageenan.

The antioxidant activity of κ-carrageenan may be related to its degree of polymerization, reduced sugar content, sulfate groups, and terminal structure [37]. Yuan et al. (2005) [38] were among the first to report that carrageenan oligosaccharides isolated from *Kappaphycus striatus* macroalgae and their super sulfated, acetylated, and phosphorylated derivatives have antioxidant activity in vitro. Suganya et al. (2016) [39] observed that carrageenan from *Kappaphycus alvarezii* macroalgae and commercial carrageenan (Sigma-Aldrich) exhibit strong antioxidant activity and the capacity to scavenge hydroxyl, nitric oxide, and DPPH radicals.

### 3.4. ATR-FTIR Spectroscopy

**Figure 6:**
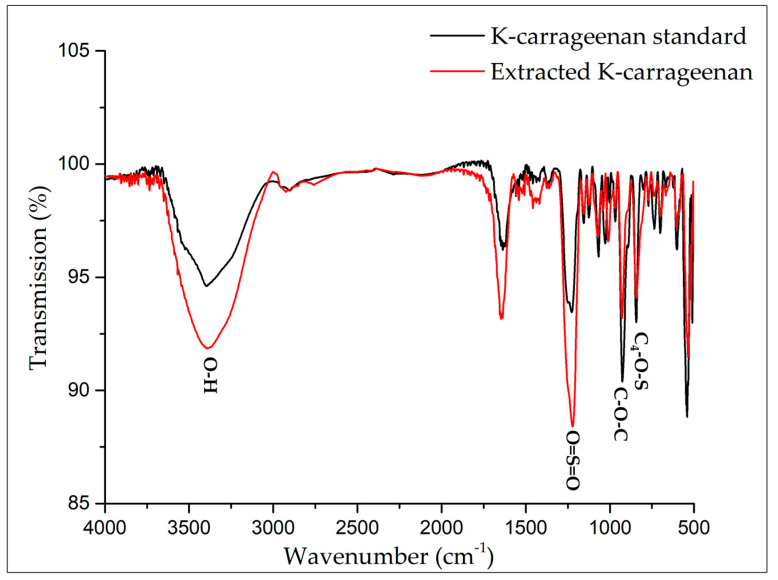
Extraction from the *G. skottsbergii* macroalgae (red) and standard κ-carrageenan (black) by ATR-FTIR analysis [42].

The ATR-FTIR spectrum of the carrageenan extracted from the *G. skottsbergii* macroalgae compared to κ-carrageenan from the Sigma standard is shown in Figure 6. Both spectra have overlapping bands at 3400, 1650, 1200, 950, 800, 700, and 550 cm^−1^. Our findings are consistent with Muthulakshmi et al. (2021) [36], who observed spectra for κ-carrageenan at 2360, 1450, 1400, 1194, 1123, 1101, 753, 656, and 601 cm^−1^.

The carrageenan spectra revealed the main features of carrageenan compared to the extracted *G. skottsbergii* carrageenan (Figure 6). Furthermore, ATR-FTIR analysis demonstrated the existence of κ-carrageenan via high absorption bands at 930 cm^−1^ (CO of 3,6-anhydrogalactose) and 845 cm^−1^ (CO-SO4 in C4 of galactose). The spectra also showed high absorption in the 1000–1100 cm^−1^ range, which is typical of polysaccharides, whereas the 1010–1080 cm^−1^ area is attributed to the glycosidic bonds found in all carrageenans. Notably, water is known to have a high absorbance at 1640–1650 cm^−1^ [40,41]. These data confirmed similar spectra, demonstrating the quality of the carrageenan. Thus, compared to the sigma κ-carrageenan standard, the ATR-FTIR results showed that carrageenan extraction from the *G. skottsbergii* macroalgae was effective.

### 3.5. κ-Carrageenan Determination by UHPLC-UV-Vis

**Figure 7:**
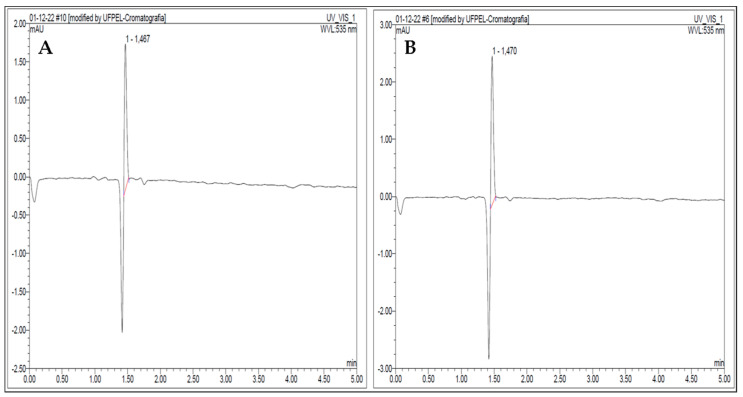
Chromatograms of the extracted carrageenan (**a**) and standard carrageenan (**b**).

The extracted sample was confirmed to be κ-carrageenan using UHPLC/UV-Vis analysis (Figure 7), which required a standard κ-carrageenan. The peaks in both chromatograms had the same chromatographic profile and retention time, indicating that the extracted sample was κ-carrageenan.

### 3.6. Binding Capacity Evaluation of G. Skottsbergii Carrageenan with SARS-CoV-2 Viral Particles

**Figure 8:**
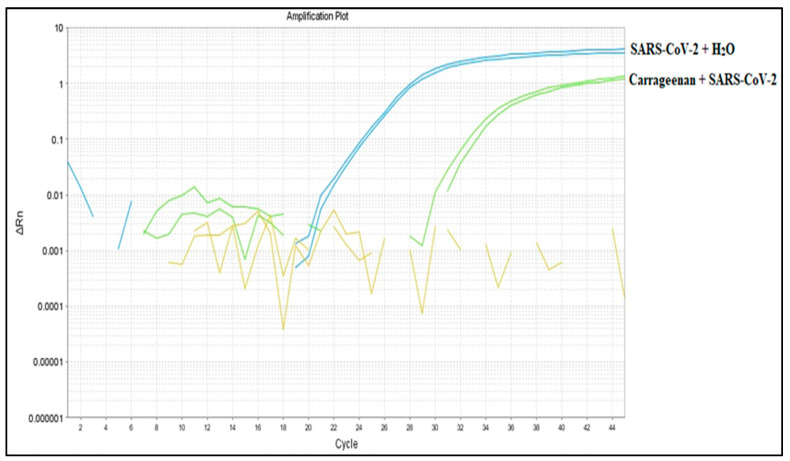
Evaluation of the binding capacity of SARS-CoV-2 with carrageenan extracted from *G. skottsbergii* macroalgae using RT-qPCR.

The RT-qPCR results in Figure 4 refer to the incubation of *G. skottsbergii* extracted carrageenan in an inactivated SARS-CoV-2 suspension during 24 h of continuous agitation. By analyzing the amplification plot (Figure 8), one can observe that the N1 gene was detected in both the control (SARS-CoV-2 + H_2_O) and tested material (carrageenan + SARS-CoV-2) samples, presenting cycle threshold (CT) values of 24.47 ± 0.15 and 32.87 ± 0.42, respectively. Nevertheless, the supernatant sample showed no detectable CT, indicating that the carrageenan captured the viral particles, likely due to the binding of carrageenan’s highly charged macromolecules to the viral surface, as suggested elsewhere [43,44,45].

The CT values are inversely proportional to the viral load [44,45] as viral DNA yield doubles at each cycle [44]. Therefore, we could estimate the viral particle’s yield in 240.392 ± 1.042 viral particles/mL for the SARS-CoV-2 + H_2_O sample and 2.002 ± 36.0 viral particles/mL for the carrageenan + SARS-CoV-2 sample. The estimated binding rate of carrageenan to viral particles was 8.3%.

**Figure 9:**
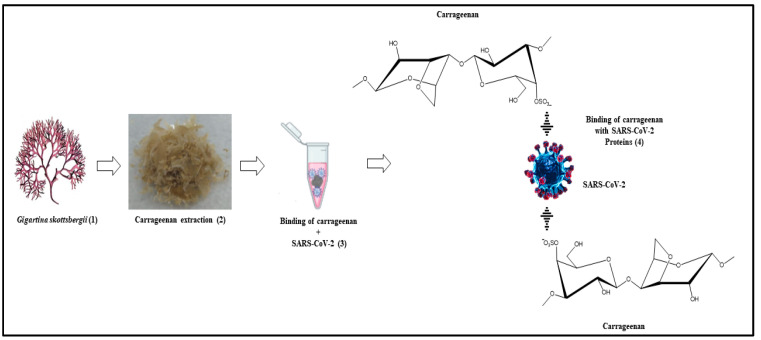
Graphical representation for SARS-CoV-2 viral particles binding in *G. skottsbergii* macroalgae (1) and the carrageenan extract (2). Binding occurs after incubation (3). Viral RNA was isolated directly from carrageenan (4).

Studies conducted throughout the COVID-19 pandemic have reported high sensibility and specificity values of RT-qPCR in COVID-19 diagnosis [31,46]. Hence, it is a highly useful tool that provides a solid basis for developing novel diagnostic and surveillance methods in clinical and environmental samples [6,23,47]. The ability of carrageenan-mediated viral RNA extraction, as illustrated in Figure 9, further emphasizes the biotechnological potential of this widely available subantarctic macroalgae. The study of Schütz et al. [48] on the prophylactic use of carrageenan-containing nasal and mouth sprays to prevent SARS-CoV-2 infection is an excellent example of the broad applicability of this biomaterial. In fact, researchers have even reported antiviral activity against other viral pathogens, such as herpes viruses [49,50] and papilloma virus [51]. Therefore, further research with carrageenan may lead to novel molecular detection methods for SARS-CoV-2 and other viruses or even improve currently available methods.

### 3.7. RT-LAMP Detection

**Figure 10:**
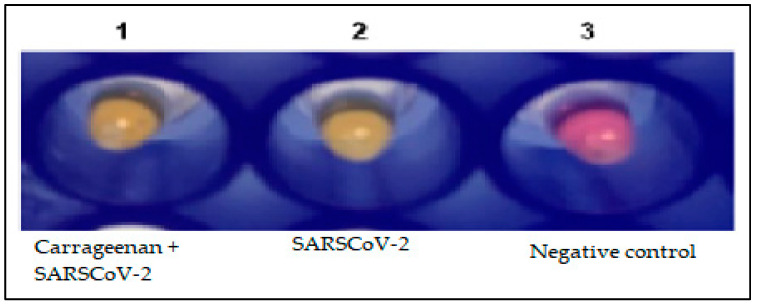
Detection of SARS-CoV-2 by the RT-LAMP assay after virus binding to carrageenan. Channel 1 and 2: positive result for SARS-CoV-2 detection. Channel 3: negative control. Yellow reaction: amplification. Pink reaction: no amplification.

In order to evaluate whether the 8.3% binding rate of carrageenan to the SARS-CoV-2 virus is applicable in diagnostic routines, we chose an alternative molecular assay called RT-LAMP. Figure 10 demonstrates the presence of viral RNA detected by RT-LAMP after binding the viral particles to carrageenan in the binding experiments in an aqueous medium (Figure 10). It is evident that carrageenan was able to sequester SARS-CoV-2 viral particles in this environment.

The RT-LAMP assay has been widely used during the COVID-19 pandemic, and we have employed it in some studies to detect SARS-CoV-2 in different clinical samples [6,52,53]; this is a more specific and less sensitive method than an RT-PCR assay to detect SARS-CoV-2.

Kitajima et al. [54] reported a high concordance rate of 93.3% between RT-PCR and RT-LAMP in sputum and a nasopharyngeal sample containing 10.2 and 23.4 copies per 10 μL, respectively. Furthermore, RT-LAMP exhibits a high degree of specificity (98.5%), sensitivity (87%), positive predictive value (97.9%), and negative predictive value (90.2%) for SARS-CoV-2 detection. Notably, limit of detection (LOD) has been reported in other studies with high sensitivity and specificity rates, with a LOD higher and lower than 100 copies/μL, as demonstrated by Cao et al. (2021) [55] and Broughton et al. (2020) [56], who found 10 and 225 copies/μL, respectively, while Zhang et al. (2021) [57] and Agrawal et al. (2020) [58] used the N gene and obtained 4 and 40 copies/μL, respectively. Our results showed a value of roughly 2000 viral copies/mL, enabling this technique to detect the viral load linked to carrageenan.

The RT-LAMP can be reliably performed, demonstrating that the 8.3% linked rate represents an excellent evolution for future applications. We believe that higher carrageenan concentrations may improve the binding rate. However, we must ensure that this increase does not interfere with transmitting the genetic material of the virus.

Another important aspect of using carrageenan prior to viral genetic material inheritance is the potential improvement in sensitivity. Its use may increase the sensitivity of molecular methods, particularly concerning borderline diagnosis; that is, in samples with low viral load, the carrageenan-SARS-CoV-2 connection could facilitate virus removal from clinical samples, and materials with small quantities of the virus could increase the probability of detection in diagnostic methods. In the COVID-19 diagnostic scenario, this would reduce the number of false negatives.

## 4. Conclusions

Carrageenan extracted from *G. skottsbergii* macroalgae demonstrated the ability to bind to viral particles of SARS-CoV-2 in an aqueous environment within 24 h of incubation. This was shown with the discovery of viral RNA isolated directly from carrageenan. The RT-LAMP test, which detected the presence of SARS-CoV-2 RNA material, validated the 8.3% binding rate. Thus, it is clear that carrageenan derived from subantarctic macroalgae has promising uses in diagnostic and therapeutic applications and various fields of biotechnology.

## Data Availability

We would like to provide data on request from the corresponding author.
